# Feed-based vaccination regime against streptococcosis in red tilapia, *Oreochromis niloticus* x *Oreochromis mossambicus*

**DOI:** 10.1186/s12917-016-0834-1

**Published:** 2016-09-08

**Authors:** M. S. Ismail, A. Siti-Zahrah, M. R. M. Syafiq, M. N. A. Amal, M. Firdaus-Nawi, M. Zamri-Saad

**Affiliations:** 1 Department of Veterinary Laboratory Diagnosis, Faculty of Veterinary Medicine, Universiti Putra Malaysia, 43400 Serdang, Malaysia; 2 National Fish Research and Disease Diagnosis, Fisheries Research Institute, 11960 Batu Maung, Penang Malaysia; 3 Department of Biology, Faculty of Science, Universiti Putra Malaysia, 43400 Serdang, Malaysia; 4 Present address: Institute of Bioscience, Universiti Putra Malaysia, 43400 Serdang, Malaysia

**Keywords:** Vaccination regime, Streptococcosis, Red tilapia, Feed-based vaccine

## Abstract

**Background:**

Streptococcosis is an important disease of tilapia throughout the world. In Malaysia, streptococcosis outbreak was commonly reported during the 3-month period of high water temperature between April and July. This study describes the duration of protection following single and double booster dose regimes against streptococcosis in tilapia using a feed-based vaccine containing formalin-killed *Streptococcus agalactiae*. A total of 510 tilapias of 120 ± 10 g were selected and divided into 3 groups. Fish of Group 1 were vaccinated at weeks 0 and 2 (single booster group) while fish of Group 2 were vaccinated at weeks 0, 2 and 6 (double booster group) with a feed-based vaccine against streptococcosis. Fish of Group 3 was not vaccinated. Serum samples were collected weekly to determine the antibody level while samples of eye, brain and kidney were collected for bacterial isolation. At week 10, all fish were challenged with live *S. agalactiae* and the survival rate was determined.

**Results:**

Both vaccinated groups showed significant (*p* < 0.05) increase in the antibody levels following the first booster dose, which lasted until week 6. Group 2 showed consistent high level of antibody following the second booster dose at week 6 and remained high until week 12. Challenge trial at week 10 resulted in 45 %, 70 % and 0 % rate of survival for Groups 1, 2 and 3, respectively.

**Conclusion:**

Double booster regime is most suitable to be applied for feed-based vaccination against streptococcosis prior to the start of the hot season.

## Background

Tilapia is one of the most cultured fish species in the world with China, Egypt and Philippines as major producers [1]. However, with extensive aquaculture activity, farmed fish are affected by environmental fluctuations and husbandry management, which cause stress to the fish and contribute to infections by a wide variety of pathogens [2].

Streptococcosis is one of the major diseases of fish that leads to staggering losses. It is caused by two main species; *Streptococcus agalactiae* and *S. iniae*, affecting a wide range of both freshwater and marine fish species. In Malaysia, most outbreaks of streptococcosis in tilapia are caused by *S. agalactiae* that are influenced by the high water temperature above 31 °C during the hot months of April to July [3].

Vaccination is the most viable option to control streptococcosis in fish [2]. Injection is the most potent route of vaccination as it produces a stronger immune response compared to other routes of vaccination such as spray and immersion [4, 5]. However, the injection method requires a certain level of manpower, technical prowess and proper equipment. Therefore, a more practical route of vaccination is the oral route as there is no direct contact between handler and fish [6]. Furthermore, no specific technical skill is needed to apply the vaccine to the fish. However, oral vaccination results in lower efficacy and shorter period of protection [2]. This paper determines the duration of systemic IgM response and efficacy of the newly developed feed-based vaccine against challenge by field strain of *S. agalactiae.*

## Methods

### The fish

Red tilapia (*Oreochromis mossambicus* x *O. niloticus*) fingerlings of approximately 2 in. in length were acquired from a hatchery in Tapah, Perak. Upon arrival at the experimental station, the fish were kept in 2 thousand litres capacity fibreglass tanks at 27 °C with running water and aerators. The fish were fed *ad-libitum* twice daily until they reached approximately 100 g bodyweight. Prior to the start of the experiment, the fish were screened for bacterial colonization and antibody level to ensure that they were free from infection by *S. agalactiae* [7].

### Bacterial culture

*Streptococcus agalactiae* strain TP 749B was used in this study. The strain was isolated from an outbreak of streptococcosis in cage-cultured red tilapia in 2007 at Sungai Pahang, Malaysia [8]. The stock that was kept in glycerol at −20 °C was thawed to room temperature overnight prior to use.

### Formalin-killed bacteria

The formalin-killed bacteria for vaccine preparation were prepared according to the method of Firdaus-Nawi et al. [6]. Briefly, stock culture of *S. agalactiae* (TP 749B) was streaked onto 5 % blood agar and incubated at 30 °C for 24 h. Propagation of *S. agalactiae* was done by inoculating 10 colonies from the blood agar into Brain Heart Infusion Broth (BHIB) and incubated in a shaker incubator at 150RPM, 30 °C for 24 h. Following incubation, the bacterial concentration was determined using the standard plate count technique [9]. The *S. agalactiae* cells were then harvested by centrifugation (6000 × g, 10 min) and washed with phosphate-buffered saline (PBS) followed by centrifugation (6000 × g, 10 min) for 3 times to remove the medium residue. Afterwards, 10 % buffered formalin was added into the washed pellet to reach a final concentration of 0.5 % formalin before the mixture was kept overnight at 4 °C to kill the bacteria. Then, the bacteria cells were washed again with PBS with centrifugation (6000 × g, 10 min) for 3 times to remove the formalin residue from the culture. The formalin-killed bacteria were re-suspended in sterile PBS solution to obtain the final bacteria concentration of 6.7 × 10^7^ CFU/mL. The suspension was streaked onto blood agar and incubated for 24 h at 37 °C to ensure that all *S. agalactiae* were killed. To improve the vaccine antigenicity, Freund’s incomplete adjuvant (FIA) was added to a final concentration of 10 % before it was thoroughly mixed with pelletted feed to give a final concentration of 10^6^ cells/kg of feed [6].

### Experimental design

A total of 510 red tilapia hybrid (*Oreochromis mossambicus* x *O. niloticus*) fish with an average weight of 120 ± 10 g were selected and equally divided into 3 groups; the single booster, double booster and control groups. Ten fish were placed into each of the 51 glass aquaria containing 100 L water at an average water temperature of 29 °C, provided with aeration and running water with 12-h light and dark periods. The fish were fed with commercial feed (Cargill, Malaysia).

At the start of the experiment, the vaccine was orally administered on day 0 by feeding the fish with feed containing the vaccine at 4 % body weight twice; at 8 am and 3 pm. To ensure that all treated fish received the feed-based vaccine, they were deprived of feed for 24 h and while feeding, the feed-based vaccine was distributed slowly and evenly in each glass aquaria. For single booster group, vaccination was repeated on day 14, while the double booster group received the feed-based vaccine on days 14 and 42. The unvaccinated control group was given regular commercial feed without the vaccine. On other days, all fish were fed with normal commercial pellet throughout the 14-week study period.

Serum samples to determine the antibody level were collected from 10 fish of each group at weekly intervals beginning from day 0 until day 98 when the experiment was terminated. The same procedure was replicated and data from both experiments were pooled and analysed.

### Challenge trial

The challenge trial was carried out at week 10 of the experiment. Twenty fish from each of the three groups were transferred into 6 glass aquaria without running water. These 20 selected fish from each group were kept in 2 aquaria at the rate of 10 fish per aquarium as duplicate. A fresh inoculum containing live virulent cells of *S. agalactiae* strain NF958 at a concentration of 1.0 × 10^9^ CFU/ml was prepared before 0.5 ml of the inoculum was injected intraperitoneal (i.p.) into each fish. The fish were anaesthetized with *Tricaine methanesulfonate* (MS-222) prior to the i.p. injection [6]. After challenged, all fish were closely observed at hourly intervals for behavioural and physical changes. Fish that died within the first 6 h were excluded from the experiment while fish that showed advanced clinical signs of the infection were euthanized. At the end of day 7 post-challenge, all surviving fish were killed by an overdose of MS-222. They were subjected to post-mortem examination before samples of brain, eye and kidneys were collected for bacterial isolation. The level of protection or relative percentage survival (RPS) value was then determined [10].

$$ \mathrm{R}\mathrm{P}\mathrm{S}=1{\textstyle -}\left(\%\mathrm{immunizedgroupmortality}/\%\mathrm{controlgroupmortality}\right)\times 100 $$

### Bacterial isolation

Organ samples were streaked onto blood agar and were incubated at 37 °C for 18 to 24 h. Following incubation dominant colonies were sub-cultured to obtain pure colonies before they were subjected to Gram staining, oxidase and catalase tests. Isolates that were Gram-positive, cocci shaped and catalase negative were subjected to API 20 STREP bacteria identification kit (BioMerieux, France). The Gram-positive cocci but catalase positive were subjected to API STAPH (BioMerieux, France). Similarly, Gram-negative isolates, which were oxidase negative, were subjected to API 20E while those that were oxidase positive were subjected to API 20NE (BioMerieux, France). The API colour codes were referred to the APILAB PLUS program (BioMerieux, France) for species and genus determination of the isolates. Cultures that were identified as *S. agalactiae* by API20 STREP were further confirmed by API 20STREP and BLAST analysis of 16S rRNA sequence of PCR product [11].

### Enzyme-linked immunosorbent assay

All serum samples were subjected to indirect ELISA [12, 13] that detected the IgM. Coating antigen was prepared by culturing *S. agalactiae* into brain-heart infusion broth and incubated for 24 h in shaker incubator at 30 °C, 150 rpm. Bacterial concentration was determined using standard plate count technique before harvested by centrifugation and washed with phosphate buffer saline (PBS). The bacterial pellet was then re-suspended in carbonate-bicarbonate buffer (pH 9.6) to a final concentration of 2.5 × 10^5^ CFU/ml. The suspension was boiled in water bath at 97 °C for 20 min to kill the bacteria and cooled to room temperature prior to use as coating antigen.

Flat-bottomed microtitre plates were coated with 100 μL coating antigen and were left overnight at 4 °C before washed twice with PBS + 0.05 % Tween 20 (PBST). Then, 200 μL of 1 % bovine serum albumin (BSA) was added to block unspecific binding sites and incubated in 37 °C for 1 h. This was followed by adding 100 μL of serum (1:1000) into the reaction and similarly incubated. After that, 100 μL of goat anti-tilapia hyperimmune serum, diluted 1:10,000 was added and incubated again. Next, 100 μL of conjugated rabbit anti-goat IgM-horseradish peroxidase (Nordic, Netherland) diluted to 1:10,000 was added into the reaction and incubated. After washed for three times with PBST, bound conjugates were detected by adding 100 μL of TMB One substrate solution (Promega, USA) before the reaction was stopped with 0.2 mol/L sulphuric acid. The absorbance was determined at 450 nm wavelength (Bio-Rad, USA).

The cut-off value is the highest possible true-positive rate [14] that is used as an indication of protection. It was determined by performing ELISA on 100 serum samples collected from non-immunized tilapia obtained from farms that had no history of streptococcosis infection and did not practice vaccination against the target organism. After subtracting the blank wells, the average OD value was times 2 to get the cut-off value of 0.2 OD.

### Statistical analysis

The significance value of the results was calculated by using one way analysis of variance (ANOVA) employed by Tukey HSD in Statistix 9 software (Analytical software, USA). The results were considered as significant at *p* < 0.05.

## Results

### Serum antibody response

**Fig. 1:**
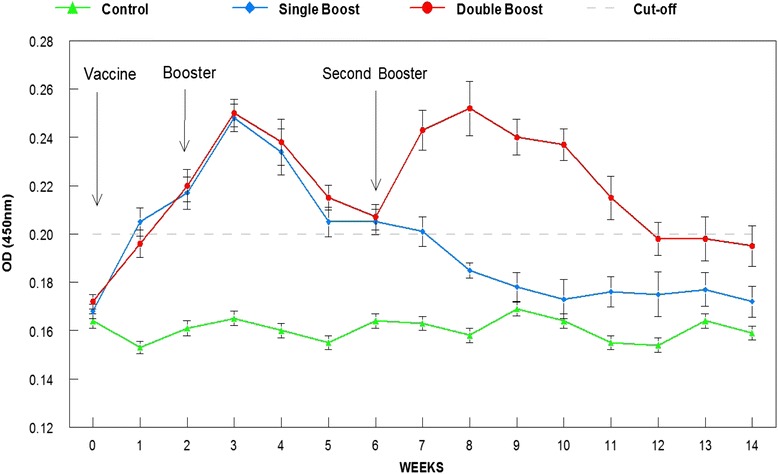
Serum antibody (IgM) response following oral vaccination with a feed-based vaccine. Double booster at weeks 2 and 6 prolonged the high antibody levels for up to 12 weeks compared to the single booster at week 2 that lasted 6 weeks

The serum IgM antibody levels of all groups prior to vaccination showed insignificant (*p* > 0.05) differences. Following vaccination, both vaccinated groups showed significantly (*p* < 0.05) higher IgM levels compared to the control group. Following the first booster dose at week 2, the increasing pattern continued for both vaccinated groups and reached peak at week 3. The level, however, started to decrease insignificantly (*p* > 0.05) in the following week but significantly (*p* < 0.05) thereafter to reach the non-protective cut-off value (*p* > 0.05) at weeks 5 and 6 (Fig. 1). In the first 6 weeks post-vaccination, both vaccinated groups showed insignificantly (*p* > 0.05) high IgM value. However, following second booster in week 6, the antibody level significantly (*p* < 0.05) increased again to reach peak at week 8 before declining thereafter until week 12 (Fig. 1). The vaccinated group that received a single booster showed gradual decline in IgM levels to reach the same level (*p* > 0.05) as the cut-off value by week 7 and below the cut-off value thereafter until the end of the experimental period. At time of challenge in week 10, the IgM level of the double booster group was significantly (*p* < 0.05) higher while the single booster and control groups were significantly (*p* < 0.05) lower than the protective cut-off value (Fig. 1).

### Challenge trial

Following intraperitoneal challenge, fish mortality was observed among the control group as early as 24 h post-challenge. Clinical symptoms were apparent in the control group, which included lethargy, exophthalmia, erratic swimming, inflammation at injection area and loss of appetite while few vaccinated fish showed signs of lethargy and loss of appetite. At the end of day 7 post-challenge, none of the unvaccinated control fish had survived compared to 45 % and 70 % survival rates of single and double booster vaccinated fish, respectively (Table 1).

**Table 1:** Comparative fish mortality, survival and relative percentage survival (RPS) between controls unvaccinated fish, fish vaccinated with single booster, and fish vaccinated with double booster regimes followed by intraperitoneal challenge with 0.5 mL of 1.0 × 10^9^ CFU/mL of live *Streptococcus agalactiae*

Group	Number of Fish	Mortality (n) Post-challenge							Survival	RPS
		D1	D2	D3	D4	D5	D6	D7	Rate (%)	
Single Booster	20	6	3	0	1	1	0	0	45	45^b^
Double Booster	20	3	2	0	1	0	0	0	70	70^c^
Control	20	7	5	3	3	2	0	0	0	0^a^

^a,b,c^Different superscripts indicate significant difference (*p* < 0.05)

Post-mortem examinations revealed congested kidney, enlarged spleen, pale liver and soft, watery brain. Haemorrhages were also registered in various organs including liver, gastrointestinal organs and brain.

### Bacterial isolation

*Streptococcus agalactiae* was successfully re-isolated from the brain, eye and kidney of all dead fish. The presence of clear zone around cultured colonies was suggestive of β-haemolytic *S. agalactiae* that was confirmed by BLAST analysis of 16S rRNA sequence of PCR product.

## Discussion

As development in aquaculture industry continues to expand, many vaccines against many diseases are made available with the purpose of controlling disease outbreaks. Commercial vaccines for tilapia against streptococcosis are currently available in many countries and are widely used. The most common type of vaccine for streptococcosis is the injectable vaccine, administered via intraperitoneal route as it provides the best protection against streptococcosis. Immersion vaccines such as AQUAVAX™ and GARVETIL™ (Intervet Pty. Ltd.) are available and are claimed to be more practical for administration but lower in efficacy [5]. The focus of these commercial vaccines, however, was mainly for large farms that can afford high manpower, technicality and facilities for carrying out such vaccinations. Small-scale private farmers in Southeast Asia such as Vietnam, Malaysia, Indonesia and Thailand, however, are unlikely to be able to afford labor-intensive vaccine protocols requiring special equipment. They, therefore, require an easier, cost effective, user friendly and less stressful method of vaccination, which is what oral vaccination can provide [14]. Furthermore, large scale fish producers can also benefit from oral vaccination since an effective, feed-based vaccine could greatly reduce stress in the production fish by avoiding handling during vaccination and lower the cost associated with manpower and equipment.

It is well documented that different routes of vaccination give different intensity of immune response and thus, different vaccine efficacy [13]. Vaccination via intraperitoneal and intramuscular injections triggers a systemic immune response with high antibody levels in a short period of time. This provides longer protection compared to immersion and oral exposure that triggers local mucosal immunity [15, 16]. Nevertheless, if sufficient amount of antigen is managed to be transported and reach the end gut segment of fish such as carp, both local and systemic immune responses are induced [17]. The second gut segment of fish especially grouper plays an important role in oral vaccination as it participates in the transportation and presentation of antigen from intestinal lumen to intra-epithelial macrophages [7]. These macrophages ingest the antigen presented by this route and remigrate from the intestine to lymphoid organs mainly the spleen and kidney before initiating systemic immune response [17]. Similar mechanisms are believed to occur in tilapia.

Our earlier study revealed that feed-based bacterin without adjuvant was able to stimulate IgM response for a brief period of 3 weeks [6]. Addition of incomplete Freund’s adjuvant was able to prolong the response to 6 weeks but was not long enough to cover the 3-month critical period. Therefore, in this study, different oral vaccination regimes were tested in trying to maintain immune response and protective efficacy to cover the 3-month critical period. Administration of second booster at week 6 resulted in a significant increase in the level of serum IgM for up to week 12, covering the 3-month period required for protection of tilapia during the hot months of April to July. This was further clarified following challenge at week 10 when 45 % and 70 % of the single booster and double booster groups survived, respectively. Therefore, double booster provided longer period of high antibody titre that protected at least 70 % of the fish. This is in agreement with earlier studies that show that a repetition of vaccine dosage or booster dose provides better immune responses and lasting protection [18, 19].

A vaccine that provides 70 % protection is considered a good vaccine, but an excellent vaccine provides >80 % protection [20]. Furthermore, oral vaccination stimulates both mucosal and systemic immune responses, which is an advantage since natural streptococcosis involves entrance of the pathogen through mucosal organs of mouth and skin before entering the blood stream [21]. Naturally, the pathogens initially encounter the mucosal barrier of skin and gut lining before reaching the blood stream. Many studies have highlighted on the presence of some key components of mucosal immunity, particularly the IgT or IgZ antibody that are associated with mucosal immunity in fish. Furthermore, the role of intestinal T cells and their functions, particularly in antigen uptake mechanisms at mucosal surfaces has been highlighted, which is important in mucosal vaccination strategies [22]. Similarly, a study in grouper revealed that oral administrations of antigen stimulate the gut-associated lymphoid tissue of the intestine leading to higher level of IgM [7].

## Conclusion

In conclusion, oral vaccination using feed-based vaccine containing formalin-killed bacteria is a viable option in tilapia vaccination against streptococcosis as it can provide systemic immune response against *S. agalactiae*. Administration of second booster dose provides longer period of protection that last for at least 12 weeks at a rate of 70 % protection.
